# Design, Development, and Optimization of Sterculia Gum-Based Tablet Coated with Chitosan/Eudragit RLPO Mixed Blend Polymers for Possible Colonic Drug Delivery

**DOI:** 10.1155/2013/546324

**Published:** 2012-11-25

**Authors:** Bipul Nath, Lila Kanta Nath

**Affiliations:** ^1^Department of Pharmaceutical Sciences, Girijananda Chowdhury Institute of Pharmaceutical Sciences (GIPS), Azara, Assam, Guwahati 781001, India; ^2^Girijananda Chowdhury Institute of Pharmaceutical Sciences (GIPS) Affiliated to Gauhati University, Azara, Assam, Guwahati 781017, India

## Abstract

The purpose of this study is to explore the possible applicability of *Sterculia urens* gum as a novel carrier for colonic delivery system of a sparingly soluble drug, azathioprine. The study involves designing a microflora triggered colon-targeted drug delivery system (MCDDS) which consists of a central polysaccharide core and is coated to different film thicknesses with blends of chitosan/Eudragit RLPO, and is overcoated with Eudragit L00 to provide acid and intestinal resistance. The microflora degradation property of gum was investigated in rat caecal medium. Drug release study in simulated colonic fluid revealed that swelling force of the gum could concurrently drive the drug out of the polysaccharide core due to the rupture of the chitosan/Eudargit coating in microflora-activated environment. Chitosan in the mixed film coat was found to be degraded by enzymatic action of the microflora in the colon. Release kinetic data revealed that the optimized MCDDS was fitted well into first-order model, and apparent lag time was found to be 6 hours, followed by Higuchi release kinetics. *In vivo* study in rabbits shows delayed *T*_max_, prolonged absorption time, decreased *C*_max_, and absorption rate constant (Ka), indicating a reduced systemic toxicity of the drug as compared to other dosage forms.

## 1. Introduction

In the recent times, colon-specific technologies have utilized single or combination of the following primary approaches, with varying degrees of success: (1) pH-dependent systems, (2) time-dependent systems, (3) prodrugs, and (4) colonic microflora-activated systems [1, 2]. Among the different approaches to achieve colon specific drug delivery system, the use of polymers specifically degraded by colonic bacterial enzymes (such as *β*-glucoronidase, *β*-xylosidase, *β*-galactosidase, and azoreductase) holds promise. Microbially activated delivery systems for colon targeting are being developed to exploit the potential of the specific nature of diverse and luxuriant microbiota associated with the colon compared to other parts of the gastrointestinal (GI) tract. These colonic microbiotas produce a large number of hydrolytic and reductive enzymes which can potentially be utilized for colonic delivery [1, 2]. Most of these systems are based on the fact that anaerobic bacteria in the colon are able to recognize the various substrates and degrade them with their enzymes. Natural gums are often preferred to synthetic materials due to their low-toxicity, low-cost, and easy availability. A number of colon-targeted delivery systems based both on combination of pH, polysaccharides and biodegradable polymers have been designed and developed by various research groups for successful delivery of drugs to the colonic region [3, 4]. Sterculia gum has not yet been used as drug carrier specifically to the colon. It is insoluble in water, hydrates quickly, and swells into a homogenious hydrogel consistency or mass which pose difficulty for its use as polysaccharide coat [5]. But, it seemed to be an interesting polymer for the preparation of hydrophilic matrix tablets [6, 7]. However, sterculia gum in the form of hydrophilic matrix cannot protect the drug from being released in stomach and small intestine. Besides, sterculia gum is expected to retard drug release due to its higher swelling index, and at the same time its degradation by the colonic microflora would make it ideal to deliver drugs in the colon. The property of higher swelling index would provide greater surface area for more bacterial enzymatic attack. This property of the gum could be used to produce hydrostatic pressure in the design of microflora triggered colon targeted drug delivery system (MCDDS). In this system, the hydrostatic force is produced by osmotic agents and polymer swelling which concurrently drives the drug out of the system through the pores created by the pore-forming agent in the inner coating after exposure of the system to the colonic fluid [8, 9]. In addition, eudragit RLPO polymer has been reported to increase the permeability to colonic fluid due to the presence of higher number of quaternary ammonium groups.

Hence, the objectives of present investigation was to design MCDDS based on swelling property of sterculia gum and to study the influence of different independent variables on dependent variables. The design of MCDDS comprises of an osmotic tablet core containing model drug azathioprine (AZA), sterculia gum as binder, and other excipients; an inner semipermeable coating which is over coated with enteric layer to provide acid and intestinal resistance. The study includes the optimization of chitosan/eudragit RLPO mixed film coating for colonic delivery of polysaccharide core and to investigate the effects of the polymer blend ratio, concentration of pore former in the coat and coating thickness on the resulting drug release and to propose the drug release mechanism of the system. The innermost layer of chitosan/eudragit RLPO provides desired intestinal resistance, but controlling drug release in the colon [10]. Eudragit L100 was deposited in order to protect the delivery system from the gastric acidic conditions. A multilayered approach was selected, since such a dosage form was less likely to undergo dose dumping, and also, it may facilitate the spreading of the drug over the inflamed regions of the colonic lumen. The feasibility of the novel MCDDS was studied using AZA as a model anti-inflammatory drug via *in vitro* evaluation of drug release characteristics and *in vivo *assessment of pharmacokinetics in rabbits [11, 12].

## 2. Materials and Methods

### 2.1. Materials

AZA was obtained as gift samples from RPG Life Sciences Pvt. Ltd, India. Commercially available sterculia gum powder was obtained from Krystal Colloids, Mumbai. Citric acid monohydrate, anhydrous lactose, magnesium stearate, disodium hydrogen phosphate (Na_2_HPO_4_), and potassium dihydrogen phosphate KH_2_PO_4_ were purchased from Loba chemie, Mumbai, India. Eudragit RLPO and Eudragit L100 were obtained from Rhom Pharm, Darmstadt, Germany). PEG 400, acetone, isopropyl alcohol and 95% ethanol, triethyl citrate, and talc were purchased from Rankem, Mumbai. *β*-glucosidase was obtained from Sigma Chemical Co, Bangalore, India. All other used chemicals were of analytical grade.

### 2.2. Microflora Degradation Studies of Sterculia Gum in Rat Caecal Contents

Microflora degradation studies of sterculia gum were conducted in phosphate buffer solution (PBS) pH 7.4 containing rat caecal content [13, 14]. Caecal contents were collected from male Wistar rats weighing 250–300 g each. The caecal contents were dispersed in PBS under anaerobic environment (bubbled with CO_2_ gas), and the concentration of the caecal contents was adjusted to 4.0, 8.0 and 12.0% (w/v) in the PBS. Finely grounded sterculia gum powder 100 mg was added into 10 mL of caecal PBS and incubated at 37°C under anaerobic condition. The pH of caecal PBS was measured at 2 h interval up to 8 h using a pH meter.

### 2.3. Preparation of Swellable Core Tablets

The detail composition of the core tablet is presented in Table 1. The core tablets of AZA having an average weight of 240 ± 5 mg were prepared by direct compression using a single stroke tablet punching machine fitted with 8 mm round standard concave punches. Sterculia gum was used as binder cum hydrophilic matrix former, anhydrous lactose as diluents, citric acid as pH regulating excipient, and magnesium stearate as lubricant [15, 16].

### 2.4. Preparation of Chitosan-Eudragit RLPO Coating Dispersions

In the initial trial, a coating solution of eudragit RLPO (10% w/v) in propan-2-ol: acetone (60 : 40) containing 15% w/w or 25% w/w concentration of chitosan was used to apply a semipermeable coat on the core tablet. PEG 400 (25% of total coating materials) was added to improve the physicomechanical property of eudragit RLPO film. The coating conditions were as follows: stainless steel pan, 200 mm diameter, four baffled, rate of rotation of the coating pan; 40 rpm, nozzle diameter of spray gun; 1 mm, spray rate; 5 mL/min, spray pressure; 2 Bar, drying temperature; 40°C [17]. After coating, the tablets were dried for 8 hours at 35–40°C in order to remove the residual solvent. 

### 2.5. Experimental Design for Coating Formulations

A full 3^2^ factorial design was used for optimization of coating solutions [18, 19]. The concentration of chitosan was selected by using central composite design (CCD) under design expert software (version 8.0). The studied factors (independent variables) were concentration of pore former, chitosan (*X*_1_), and weight gain in coating thickness, eudragit RLPO (*X*_2_). The dependent variables selected for the study include lag time for drug release up to 2% in SCF (*Y*_1_) and percent drug release in 12 hours (*Y*_2_) and 18 hours (*Y*_3_).

### 2.6. Physical Evaluation of the Coated Tablets under Factorial Design

The thickness, hardness, drug content uniformity and weight uniformity were determined in a similar manner as stated for conventional oral tablets in the accredited pharmacopoeia.

### 2.7. *In Vitro* Dissolution Study of Chitosan-Eudragit RLPO Coated Tablets

In order to optimize the coating formula containing different concentration of pore former, *in vitro* dissolution studies of core coated with different proportions of coating materials were carried out in USP dissolution test apparatus, Type I (Campbell Electronics, Mumbai, India) in 900 mL of simulated colonic fluid (SCF is phosphate buffer medium, pH 7.4 containing rat caecal content 4% and *β*-glucosidase 2% w/v) for 18 hours under anaerobic environment [20, 21]. *β*-glucosidase was added to degrade chitosan, in colonic environmental conditions. Aliquots of dissolution fluid were analyzed at specified time intervals to determine the release of AZA by UV-visible spectrophotometer at wavelength of 281 nm. 

### 2.8. Statistical Analysis of Data and Coating Optimization

The response values (lag time in hour, % drug release in 12 hour and 18 hour resp.) of coated tablets based on 3^2^ factorial design were subjected to analysis by response surface reduced quadratic model with the help of Design Expert software (Version 8.0). Statistical validity of the polynomial was established on the basis of ANOVA provision in the design expert software, and significant terms (*P* < 0.05) were chosen for final equations [18, 22]. Response surface plots and 3D contour plots were constructed using the output files generated.

### 2.9. Enteric Coating of Chitosan-Eudragit RLPO Coated Tablets with Eudragit L100

Eudragit L100, which dissolves above pH 6.0, was selected for enteric coating [23]. The optimized chitosan-eudragit RLPO coated tablets were further over coated with enteric coating using 10% w/v of eudragit L100 in 95% ethanol. The total weight gain of eudragit L100 coating was 10% w/w. Eudragit L100 was dissolved in 95% ethanol under high stirring condition until a clear solution was obtained. Triethyl citrate (TEC), 10% w/w of total dry polymer was added as plasticizer and talc (1.5% w/w of dry polymer) as a glidant. The coating conditions were same employed under semipermeable coating.

### 2.10. Kinetic Evaluation of Drug Release Data and Stability Studies

Dissolution data of the optimized formulation was fitted to various mathematical models in order to describe the mechanism of drug release [24, 25]. The corelation coefficient (*r* ^2^) was taken as the criteria for choosing the most appropriate model. The selected formulations were tested for a period of 8 weeks at different storage conditions of 25°C and 40°C with 60% RH and 75% RH, to evaluate their drug content, hardness, and *in vitro* dissolution rate [26].

### 2.11. HPLC Assay

In the present method, the plasma 6-mercaptopurine (6-MP) rather than AZA concentration was measured because after oral administration AZA is quickly converted into its active metabolite 6-MP. The 6-MP concentration in plasma was determined according to the HPLC method reported by Shao-Jun et al. [27]. The HPLC system consisted of a Rheodyne Isocratic pump (Model-LC-10, Shimadzu Corp., Kyoto, Japan) a model 2250 pump (Bischoff, Germany), and a UV detector (Model-SPD, Shimadzu Corp., Kyoto, Japan) set at a wavelength of 325 nm (*λ*_max_). The samples were chromatographed on a reverse phase Hypersil ODS C18 column (5 *μ*m, 25 cm × 4.6 mm i.d., Thermo Electron Company, Bellefonte, North America) protected with a guard column (40 × 4 mm) packed with the same material. The mobile phase was consisting of 80 parts of 0.01 M KH_2_PO_4_ and 20 parts of Acetonitrile (80 : 20, v/v, pH 4.5). It was pumped at a flow rate of 1 mL/min for the run time of 10 min under the experimental conditions with an injection volume of 20 *μ*L [28, 29]. The column was thermostated at an ambient temperature 30°  ± 2°C throughout the study. The effluent was monitored with the UV-Visible detector at 325 nm. Metronidazole was used as an internal standard (IS).

### 2.12. *In Vivo* Study in Rabbits

The pharmacokinetics of marketed tablet (MKT), enteric coated tablet (EC), and MCDDS of AZA were assessed and compared in rabbits in a randomized, two-period crossover study. The washout period between administrations was one week. Six rabbits each weighing from 1.5 to 2.0 kg were used in this study. The rabbits were fed standard laboratory chew diet with water and fasted overnight before the experiments. The animals used in the experiments received care in compliance with the “Principles of Laboratory Animal Care” and “Guide for the Care and Use of Laboratory Animals.” Experiments followed an approved protocol from Department of Pharmaceutical Sciences, Dibrugarh University Institutional Animal Ethical Committee.

The MKT, EC, and MCDDS (containing 50 mg/Kg of drug) were orally administered in rabbits. At time intervals, two milliliters of blood samples were collected from marginal ear vein into heparinized tubes and centrifuged at 5000 rpm for 15 min at 4°C to separate plasma. The plasma samples, 0.2 mL, were deproteinized with 2.0 mL of methanol and acetonitrile mixture (1 : 1, v/v), vortexed for 5 min, centrifuged at 6000 rpm for 15 min, and supernatants were collected. The supernatants were evaporated to dryness under a gentle nitrogen stream at 40°C. The residues were reconstituted in 200 *μ*L of mobile phase, and then 20 *μ*L of each solution was injected into the HPLC column for analysis of the drug *in vivo.* Blood sampling time points were 0, 1, 2, 3, 4, 5, 6, 7, 8, 9, 10, 12, 14, 16, 18, 20, 22, and 24 hours after administration of the EC and MCDDS. For the MKT tablet of AZA, blood samples (2.0 mL) were drawn at 0, 0.5, 1, 2, 4, 5, 6, and 24 h after administration. The drug concentration of plasma samples was determined using a validated HPLC procedure as described by Shao-Jun et al. [27].

### 2.13. Determination of Pharmacokinetic Parameters and Data Analysis

Pharmacokinetic parameters were calculated by noncompartment analysis based on statistical moment theory using Microsoft Excel software. The pharmacokinetic parameters, such as maximum plasma concentration (*C*_max_) and time of maximum concentration (*T*_max_), were obtained directly from the plasma concentration-time plots. The area under the plasma concentration-time curve up to the last time (*t*) (AUC_0−*t*_), area under curve extrapolated to infinity (AUC_0−*∞*_) and area under the first moment curve extrapolated to infinity (AUMC_0−*∞*_) were calculated using the linear trapezoidal rule. The mean residence time (MRT) was calculated as AUMC/AUC. Results were expressed as mean ± standard deviation. Variations in pharmacokinetic parameters were tested using analysis of variance (ANOVA). In all the cases, a value of *P* < 0.05 was considered statistically significant.

## 3. Results and Discussion

### 3.1. Microflora Degradation Studies of Sterculia Gum

**Figure 1:**
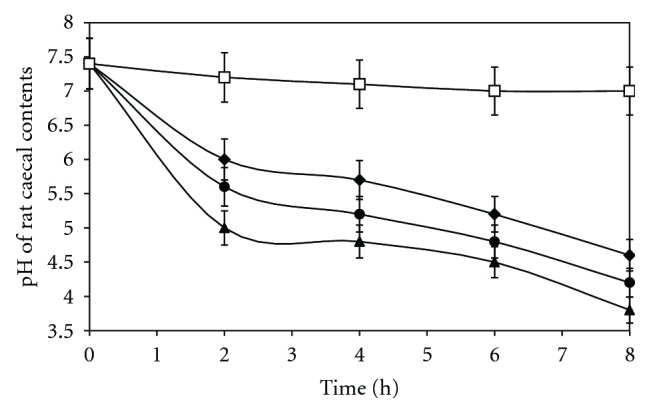
Changes of pH of phosphate buffer medium containing rat caecal content (–) 4%, (–) 8%, (–) 12% with and without sterculia gum (–). Each point represents the mean ± SD.

Microflora degradation studies of sterculia gum revealed that the pH of caecal-PBS was decreased markedly from pH 7.4 to 5.0 after incubation for 2 h with sterculia gum. The rate of decrease of pH was depended on the concentration of caecal contents within the 8 h of incubation (Figure 1). The decrease in pH was due to the appearance of degradation products of sterculia gum such as organic acids by the bacterial enzyme present in rat caecal contents.

### 3.2. Formulation Aspects of Core Tablets

The weight of each tablet was determined to be within the range of 240 ± 5 mg in order to maintain the relatively constant volume and surface area. The core tablet (240 mg each) was prepared at average tensile strength of 4.0 Kg/cm^2^ and average diameter of 8 mm and thickness 4 mm. The incorporation of citric acid in the core composition increased the hydration of large amount of the gum and expanded its volume to great extent.

### 3.3. Evaluation of the Chitosan/Eudragit Coated Tablets

The weight variation was in the range of 275 ± 2.09 to 287 ± 1.98 mg and friability was less than 0.5%. Uniformity in drug content was found among different batches of the tablet, and the drug content was more than 95%.

### 3.4. Influence of Coating Formulation Variables on Drug Release

The core tablet was successfully coated by conventional pan coating technique with varying proportion of chitosan-eudragit RLPO provided by central composite design. The coating composition of the various formulations under 3^2^ factorial designs are presented in Table 2. The results of the *in vitro* dissolutions studies of different batches of coated tablets indicated that increase in concentration of chitosan from 15% to 25% w/w and keeping constant weight gain in thickness of polymers at 10% w/w, the lag time (the time required for drug release up to 2% in SCF) was significantly decreased from 0.60 h to 0.25 h (FC1 < FC4 < FC7). The lag time was determined by separately running dissolution studies of chitosan/eudragit coated tablets in SCF for one hour at minimum time intervals. The amount of chitosan present in the eudragit coat was the key factor for such lag time. Lower amount of chitosan shows longer lag time, and higher amount shows shorter lag time.

### 3.5. Effects of Concentration of Chitosan on Drug Release

**Figure 2:**
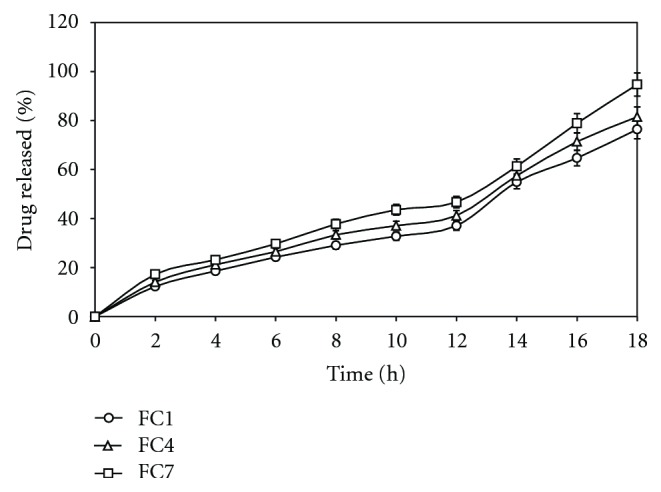
Effect of increasing concentration of pore former, chitosan (FC1-15%) (FC4-20%) and (FC7-25%) on* in vitro* drug release in SCF. Each point represents the mean ± SD.

To study the effect of concentration of chitosan, its concentration in the coating solution was kept at 15% w/w for the batch FC1, 20% w/w for FC4, and 25% for FC7. The result of the *in vitro* release profile from these formulations is shown in Figure 2. It is observed that concentration of chitosan has direct effect on drug release. The formulation FC7 containing highest concentration (25% w/w) of chitosan in the coating composition released more than 90% of AZA after 18 h of the dissolution study. This might be due to the reason that an increased in the amount of chitosan (FC7 > FC4 > FC1), it became more susceptible to bacterial attack creating pores immediately resulting in shorter lag time (0.15 h) for drug release. 

### 3.6. Effect of % Weight Gain in Coating Thickness

**Figure 3:**
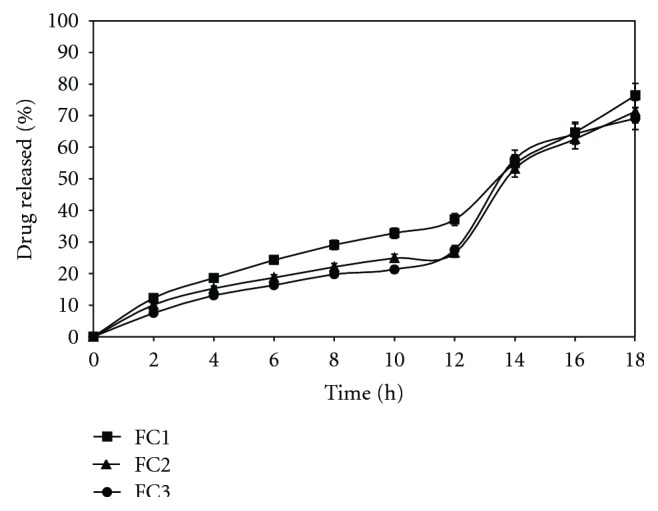
Effect of increasing coating thickness of eudragit RLPO (FC1-10% w/w), (FC2-12% w/w) and (FC3-14% w/w) on *in vitro *drug release in SCF. Each point represents the mean ± SD.

It was observed that increased in the level of weight gain from 10%, 12%, and 14% in the batches of FC1, FC2, and FC3 and keeping the concentration of chitosan constant at 15% w/w made chitosan particles less susceptible to bacterial attack, resulting in longer lag time and lesser percentage of drug released in 18 h owing to less accessibility of the chitosan particles across the eudragit coat by the colonic bacteria.  Figure 3 shows that as the coating thickness was increased, drug release was decreased, as evidenced by the difference factor *f*1 value which was lower than 15. For the calculation of *f*1 and *f*2 (similarity factor) values, only one data point at which more than 85% of the drug release had been released was taken into consideration. Drug release decreased.

### 3.7. Statistical Analysis of Dissolution Data

ANOVA of the dependent variables indicated that the assumed regression models were significant (*P* < 0.0001) and valid for each considered response (Table 3). The response values of the coated tablets based on factorial design generated a mathematical model, which indicated that both the level of pore former and coating thickness had significant influence on percentage of drug release in the simulated colonic fluid at pH 7.4. The equations of the responses were found to be as follows:

(1) Y1  =0.30−0.12  X1+0.094  X2+0.025  X1  X2  +0.069  X12+0.019  X22Y2=47.91482+0.93240  X1−2.5969  X2,Y3=72.42394+1.44879  X1−1.69934  X2.

**Figure 4:**
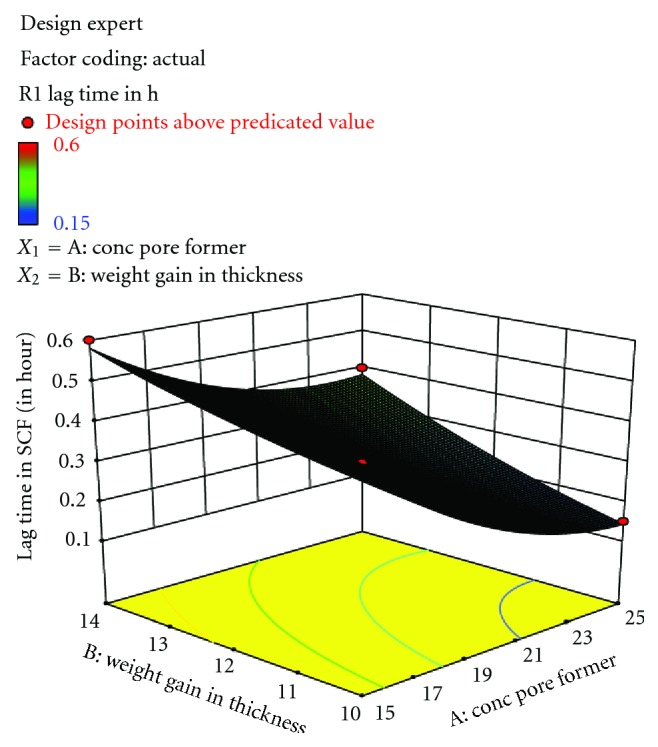
Response surface plot for response showing the influence of concentration of chitosan and % weight gain in thickness on lag time.

The above second-order polynomial equations represent the quantitative effects of independent variables (*X*_1_ and *X*_2_) upon the responses (*Y*_1_, *Y*_2_, and *Y*_3_). The validity of the above equations was justified by substituting the values of *X*_1_ and *X*_2_ in (1) to obtain the predicted values of *Y*_1_, *Y*_2_, and *Y*_3_. The observed and predicted values for the *Y*_2_ response were found to be in good agreement (Table 4). The three-dimensional response surfaces plots were drawn to estimate the effects of the independent variables on each considered response (Figure 4).

### 3.8. Optimization of Chitosan-Eudragit RLPO Coating

The best colonic drug delivery system based on coating with microporous eudragit RLPO containing optimum amount of chitosan would be a system that could protect drug release in the higher parts of the small intestine and deliver the drug only at the colonic region. Chitosan particles in the RLPO coat remained undigested in the intestinal fluid due to absence of bacterial enzyme, but degraded in the colonic fluid due to the presence of vast anaerobic bacteria and allowed the drug release to occur. Therefore, the concentration of chitosan in the eudragit coat could be the key factor for lag time. The lag time was inversely related to the level of chitosan in the eudragit coat. The lag time in colonic environment (pH 7.4) was considered as response *Y*_1_ and optimum duration for the response was considered to be 30 minutes. During this lag time, the chitosan in the eudragit coat comes in contact with the colonic bacteria formed *in situ* delivery pores for release of the drug. Thus, the percent of drug release in 12 h and 18 h was considered as response *Y*_2_ and *Y*_3_ with a constraint of minimum of 40% and 80% release, respectively. A suitable formulation which could meet these target responses would be able to release the maximum amount of drug in the colon despite its 2 h lag time in simulated gastric fluid (SGF, 0.1 M HCl at pH 1.2 containing 3.2 mg/mL pepsin) and 4 h lag time in simulated intestinal medium (SIF, phosphate buffer media at pH 6.8 containing 5 mg/mL pancreatin). 

**Figure 5:**
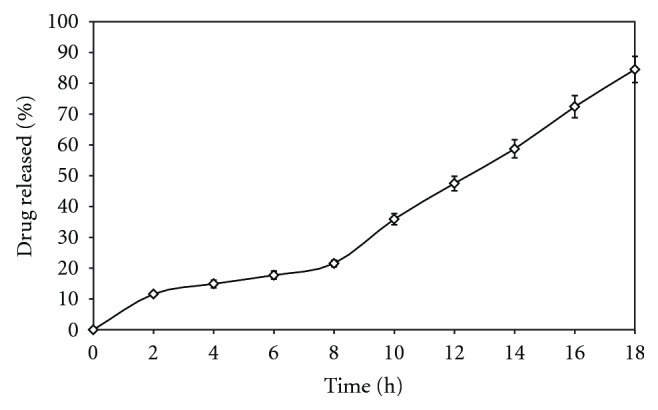
Dissolution rate profile of optimized chitosan-eudragit RLPO coated tablet in SCF.

The best formulation showing drug release corresponded to 18.96% of chitosan (pore former) and 11.3% of coating thickness of eudragit RLPO film provided the desired release as shown in Figure 5. The above quantity (*X*_1_ and *X*_2_) of formulation was substituted in (1) to obtain the predicted responses. The validity of the optimization procedure was confirmed by preparing a new batch of coating formulation with the concentration provided by the software and the observed response were found to be inside the constraints and close to the predicted responses. Thus, the factorial design was valid for predicting the optimum formulation.

**Figure 6:**
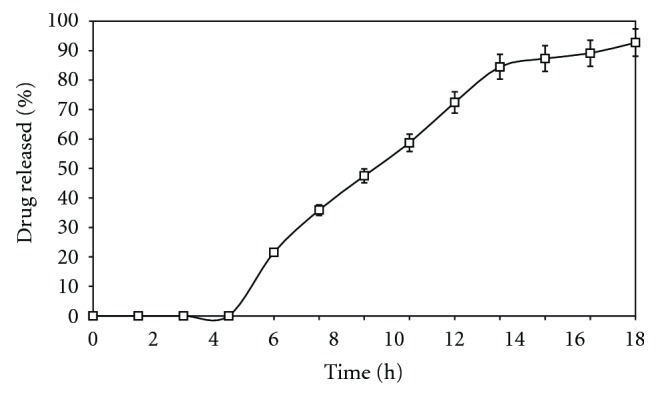
Dissolution rate profile of optimized MCDDS under continuous dissolution rate test in different media (0–2 h in SGF at pH 1.2, 2–6 h in SIF at pH 6.8 and the rest of experiment in SCF at pH 7.4).

Results of *in vitro* dissolution study showed that the over coating with 10% w/w of enteric coating material (eudragit L100, dissolves above pH 6.0) provided the desired acid and intestinal resistance of the optimized chitosan-eudragit RLPO coated tablet.  Figure 6 shows the *in vitro* release profile of optimized MCDDS in sequential phosphate buffer medium at different pH releasing more than 90% of the drug within 24 h duration.

### 3.9. Mechanism of Drug Release from MCDDS and Stability

Release kinetic data revealed that the optimized MCDDS was fitted well into first-order model and apparent lag time was found to be 6 hour, followed by higuchi spherical matrix release. It was evident that *r* ^2^ (0.9888) value was higher in first-order kinetic model as compared to the other release models. The reason for first-order kinetic release was due to the presence of enzyme degradable chitosan in the eudragit RLPO film which led to the formation of *in situ* orifices by bacterial enzyme and leaching out drug into the surrounding medium from the central polysaccharide core tablet containing sterculia gum. When the majority of chitosan particle in the eudragit coat was degraded by colonic bacterial enzymes, it ruptured due to swelling pressure of the gum core and a gradual increase in drug release was observed, as swelling increases greater surface area of sterculia gum available for bacterial action. From the stability study, the developed MCDDS was found to bestable, because there was no significant change in the percentage drug content and hardness after six month of stability study stored at 40°C ± 2°C/75%  ±  5% RH.

### 3.10. HPLC Method Development

A novel simple, precise, selective, specific, reproducible, and low cost routine reverse phase HPLC method was developed and validated as per ICH guidelines. There were no such interfering peaks observed between the retention time of 6-MP and IS. A good resolution was obtained between 6-MP and IS with retention time of 7.88 minutes for 6-MP and 4.9 minutes for IS. The method was found to be linear (*r* ^2^ = 0.999) within the analytical range of 53.32 to 4975.00 ng/mL. Maximum recovery of the drug was obtained by using methanol: acetonitrile mixture (1 : 1). The results of the method validation were proved to be accurate and reproducible, and the drug was stable in rabbit plasma up to one month period at room temperature and at three freeze-thaw cycles.

### 3.11. *In Vivo* Evaluation of MCDDS

**Figure 7:**
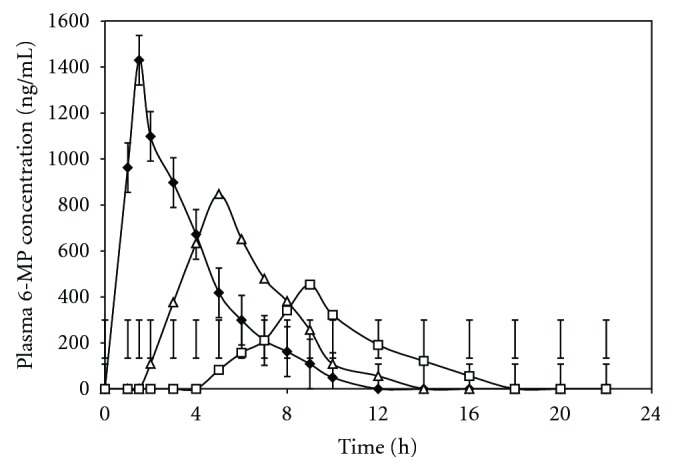
Comparison of mean plasma drug concentration versus time profile curve after oral administration of MKT tablet (–), EC tablet (▵) and MCDDS (–) of AZA in rabbit.

Mean plasma 6-MP concentration *versus *time profiles after a single oral dose of MKT, EC, and MCDDS are depicted in Figure 7. Mean values of pharmacokinetic parameters are summarized in Table 5. In case of, MKT, the peak plasma concentration (*C*_max_) of 6-MP was obtained within 1.5 h of administration, indicating the immediate absorption of AZA from the gastrointestinal tract and quick conversion into its active metabolite, 6-MP in blood. The *C*_max_ value of 6-MP following oral administration of MKT tablet was found to be 1430.08 ng/mL at the time maximum (*T*_max_) of 1.5 h. The *C*_max_ value of 6-MP for EC tablet of AZA without containing sterculia gum was found to be 847.5 ng/mL at *T*_max_ of 5.0 h. From the results of *in vitro* release study, it was observed that the drug was released after 2.0 h of dissolution study which was quite desirable, due to the fact that the drug would be released from the tablets after passing the stomach region as the tablets were enteric coated. The results of *in vivo* studies of EC tablets showed that drug was not released in the stomach up to 2.0 h and therefore it gives *T*_max_ of 5.0 h. Thus, the *in vivo* finding has good correlation with the *in vitro* results. A lag time of 6.0 h was observed from the MCDDS which revealed that the tablet had passed through the GIT and after reaching the colon only the drug was released and appeared in plasma as 6-MP. Therefore, the *C*_max_ value of 6-MP for the MCDDS could found to be 453.56 ng/mL at *T*_max_ of 9.0 h after oral administration. The results of ANOVA revealed that there was significant difference of AUC_0-∞_ between the MCDDS, EC and MKT formulation (*P* < 0.05). The results explained that the MKT formulation was more rapidly absorbed from the upper gastrointestinal tract of rabbit. But the EC and MCDDS were not absorbed from the upper GIT due to which they showed greater value of AUC_0−*∞*_ as shown in the Table 5. It is evident that AUC for MCDDS was higher as compared to the reference formulation EC and MKT formulations (MCDDS < EC < MKT). Result suggests that the extent of absorption of AZA from the developed MCDDS was decreased from the large intestine, but increased from the upper part of the GIT as seen in case of EC and MKT formulation. From the* in vivo *studies, the *C*_max_ of MCDDS was found to be almost half of the EC tablet without containing sterculia gum. The longer *T*_max_ value (9.0 h) and low *C*_max_ value (453.56 ng/mL) of MCDDS as compared to the reference formulations had proved that the MCDDS released drug only at the colonic region of the rabbit intestine. This reveals localization of the drug in the colonic mucosa from the MCDDS and thereby, possibly reducing the risk of systemic toxicity. 

## 4. Conclusions

Microflora degradation study revealed that sterculia gum can be used to release drug in the colonic region by utilizing the action of enterobacteria. The developed MCDDS exhibit gastric and small intestinal resistance but were susceptible to bacterial enzymatic attack and the potential of the system as a carrier for drug delivery to the colon is confirmed. The swelling property of sterculia gum can be used to produce hydrostatic pressure inside the tablet if it is coated with semipermeable membrane and can be used to target drug to the colon. Chitosan-eudragit RLPO mixed film coating provided the favourable characteristics to the sterculia gum core tablets to deliver it directly into the colon. Chitosan in the mixed film coat was found to be degraded by enzymatic action of the microflora in the colon. The degradation of chitosan was the rate-limiting factor for drug release in the colon. Drug release from the MCDDS was directly proportional to the concentration of chitosan, but inversely related to the weight gain in thickness of eudragit RLPO coat. The enteric layer of eudragit L100 could protect eudragit RLPO membrane containing chitosan from formation of pore or rupture before SCF dissolution procedure. Drug release from optimized MCDDS fitted well into first-order kinetic model followed by higuchi spherical matrix release model. The HPLC method developed shows good resolution to evaluate the pharmacokinetic parameters of the drug. Pharmacokinetic studies revealed that the MRT value (13.81 h) was higher for MCDDS as compared to the other two reference formulations, which were 3.60 h for MKT and 6.62 h for EC tablets, respectively. Finally, *in vivo* evaluation of MCDDS in rabbit showed delayed *T*_max_, prolonged absorption time, decreased *C*_max_, and decreased absorption rate constant (Ka) indicating that drug was slowly absorbed from the colon making the drug available for local action in the colon, thereby, reducing the risk of systemic toxicity of the drug as compared to other dosage forms.

## Figures and Tables

**Table 1:** Composition of the swellable core tablet.

Ingredients	Weight in % w/w
Azathioprine	20.83
Sterculia gum	35.83
Citric acid	15.15
Anhydrous lactose	26.19
Magnesium stearate	2.0

Total	100

**Table 2:** Composition of the chitosan-eudragit RLPO film coating.

Coating materials (% w/w)	Formulation code								
	FC1	FC2	FC3	FC4	FC5	FC6	FC7	FC8	FC9
Micronized chitosan	15	15	15	20	20	20	25	25	25
Eudragit RLPO	60	60	60	55	55	55	50	50	50
PEG 400	25	25	25	25	25	25	25	25	25
Total weight	100	100	100	100	100	100	100	100	100
% Weight gain in thickness	10.0	12.0	14.0	10.0	12.0	14.0	10.0	12.0	14.0

**Table 3:** ANOVA of dependent variables of chitosan/eudragit RLPO coated tablets.

Sources of variation	Sum of squares	*DF*	Mean square	*F*-ratio	Prob > *F *
					*P* value
*Y*1 (lag time in SCF)					
Regression	0.23	5	0.046	212.17	<0.0001
Residuals	0.00	7	0.00		
Total	0.23	12	0.46		

*Y*2 (% rel in 12 h)					
Regression	389.68	2	194.84	116.67	<0.0001
Residuals	16.70	10	1.67		
Total	406.38	12			

*Y*3 (% rel in 18 h)					
Regression	512.21	2	256.10	89.29	<0.0001
Residuals	28.68	10	2.87		
Total	540.89	12			

**Table 4:** Predicted and observed value of experimental coating formulations.

Coating formulations	Factor *X*_1_	Factor *X*_2_	Observed	Predicted	Residual
FC1	15.00	10.00	37.10	35.99	1.11
FC2	12.93	12.00	26.50	28.88	−2.38
FC3	15.00	14.00	27.50	25.63	1.87
FC4	20.00	9.17	41.20	42.7	−1.5
FC5	20.00	12.00	35.60	35.47	0.13
FC6	20.00	14.83	27.80	28.14	−0.34
FC7	25.00	14.00	38.70	32.81	5.89
FC8	27.07	12.00	41.70	42.05	0.35
FC9	25.00	10.00	46.70	44.50	2.20

**Table 5:** Comparison of pharmacokinetic parameters obtained after oral administration of three different formulations of AZA in rabbits with an equivalent dose of 50 mg/Kg.

Pharmacokinetic	MKT tablet of AZA	EC tablet of AZA	MCDDS
parameters			
AUC_0–*∞*_	2130.76	2678.13	3380.41
*C*_max_ (ng/mL)	1430.08	847.5	453.56
*T*_max_ (h)	1.50	5.00	9.00
Ke (h^−1^)	1.024	0.0911	0.0501
(*t*_1/2_)^e^ (h)	0.6820	7.59	12.95
AUMC_0–*∞*_ (h^2^·ng/mL)	7670.73	17729.22	46677.81
MRT (h)	3.60	6.62	13.81
Ka (h^−1^)	3.18	5.6	11.8
(*t*_1/2_)^a^ (h)	0.2179	0.1239	0.0587
*V*_*d*_ (L)	2.26	7.80	12.71
TCR (L/h/kg)	2.31	0.7105	0.6367
